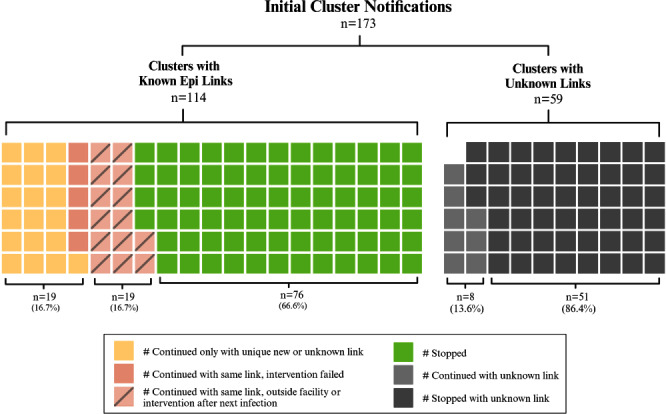# Real-time Whole Genome Sequencing Surveillance as an Effective Outbreak Detection and Mitigation Tool

**DOI:** 10.1017/ash.2024.269

**Published:** 2024-09-16

**Authors:** Alexander Sundermann, Marissa Griffith, Kady Waggle, Vatsala Rangachar Srinivasa, Nathan Raabe, Deena Ereifej, Hunter Coyle, Rose Patrick, Ashley Ayres, Daria Van Tyne, Lora Pless, Graham Snyder, Lee Harrison

**Affiliations:** University of Pittsburgh; UPMC; University of Pittsburgh School of Public Health, Department of Epidemiology

## Abstract

**Background:** Detection of outbreaks traditionally relies on passive surveillance, and often misidentify or miss outbreaks. Whole genome sequencing (WGS) surveillance has emerged as a proactive measure, enabling early detection of outbreaks and facilitating rapid intervention strategies. WGS surveillance has not been widely studied due to infrastructure, cost, and evidence barriers regarding its impact on reducing healthcare-associated infections (HAIs). This study represents findings from two years of a real-time WGS surveillance program called the Enhanced Detection System for Healthcare-associated Transmission (EDS-HAT). **Methods:** The study was conducted at UPMC Presbyterian hospital, a 694-bed tertiary care center. Patient isolates of select bacterial pathogens were collected and underwent WGS weekly from November 2021 to November 2023. Potential transmission was defined using single-nucleotide polymorphism thresholds (≤15 for all organisms except Clostridioides difficile). Genetically related clusters were reviewed weekly for epidemiological linkages (unit, personnel, or procedural commonalities) and appropriate interventions were initiated by the infection prevention and control team. We described the frequency of genetic relatedness and nature of epidemiological linkages. **Results:** Of 7,051 eligible unique patient organism isolates, 4,723 were deemed healthcare-associated and underwent WGS. EDS-HAT identified 478 (12.2%) isolates genetically related to ≥1 other isolate across 173 clusters. Epidemiological links were found in 278 (58.2%) isolates in 114 clusters, with the majority being unit-based (205 isolates, 71.9%); other epidemiological links included equipment or healthcare workers (32 isolates, 11.5%), external facilities (24 isolates, 8.6%), and shared endoscopes (17 isolates, 6.1%); all endoscope outbreaks were effectively contained at two patients. No epidemiological links could be identified for 200 (41.8%) isolates. Infection prevention initiated 134 interventions in 114 clusters, including 74 (55.2%) general staff notification and education, 25 (18.7%) enhanced cleaning efforts, 23 (17.2%) hand hygiene/personal-protective equipment compliance observations, 9 (6.7%) environmental cultures, and 3 (2.2%) enhanced microbiological surveillance. Following the detection of an epidemiological link and intervention, 94/101 (94.1%) outbreaks were effectively halted on the intervened route (Figure). **Conclusion:** This study demonstrates the feasibility and efficacy of EDS-HAT as an infection prevention tool. Early detection and intervention of outbreaks significantly enhance the capability of healthcare facilities to control and prevent the spread of HAIs. Investment in infrastructure and implementation costs will result in reducing pathogen transmission and improving patient safety in acute care settings.

**Disclosure:** Alexander Sundermann: Honoraria - Opgen

**Figure:**